# In vitro corrosion of ZEK100 plates in Hank's Balanced Salt Solution

**DOI:** 10.1186/1475-925X-11-12

**Published:** 2012-03-13

**Authors:** Hazibullah Waizy, Andreas Weizbauer, Christian Modrejewski, Frank Witte, Henning Windhagen, Arne Lucas, Marc Kieke, Berend Denkena, Peter Behrens, Andrea Meyer-Lindenberg, Friedrich-Wilhelm Bach, Fritz Thorey

**Affiliations:** 1 Department of Orthopedic Surgery, Hannover Medical School, Anna-von-Borries-Str.1-7, 30625 Hannover, Germany; 2 Institute of Production Engineering and Machine Tools (IFW), Leibnitz University of Hannover, Lise-Meitner-Str. 1, 30823 Garbsen, Germany; 3 Institute for Inorganic Chemistry, Leibniz University of Hannover, Callinstr. 9, 30167 Hannover, Germany; 4 Small Animal Clinic, School of Veterinary Medicine Hannover, Bischhofsholer Damm 15, 30173 Hannover, Germany; 5 Clinic for Small Animal Surgery and Reproduction, Centre of Clinical Veterinary Medicine, Faculty of Veterinary Medicine Ludwig-Maximilians-Universität München, Veterinärstr. 13, 80539 Munich, Germany; 6 Institute of Materials Science, Leibniz University Hannover, An der Universität 2, 30823 Garbsen, Germany; 7 Center for Hip, Knee and Foot Surgery, ATOS Clinic Heidelberg, Bismarckstr. 9-15, 69115 Heidelberg, Germany

**Keywords:** Magnesium alloy, Corrosion, Plates, in vitro study

## Abstract

**Background:**

In recent years magnesium alloys have been intensively investigated as potential resorbable materials with appropriate mechanical and corrosion properties. Particularly in orthopedic research magnesium is interesting because of its mechanical properties close to those of natural bone, the prevention of both stress shielding and removal of the implant after surgery.

**Methods:**

ZEK100 plates were examined in this in vitro study with Hank's Balanced Salt Solution under physiological conditions with a constant laminar flow rate. After 14, 28 and 42 days of immersion the ZEK100 plates were mechanically tested via four point bending test. The surfaces of the immersed specimens were characterized by SEM, EDX and XRD.

**Results:**

The four point bending test displayed an increased bending strength after 6 weeks immersion compared to the 2 week group and 4 week group. The characterization of the surface revealed the presence of high amounts of O, P and Ca on the surface and small Mg content. This indicates the precipitation of calcium phosphates with low solubility on the surface of the ZEK100 plates.

**Conclusions:**

The results of the present in vitro study indicate that ZEK100 is a potential candidate for degradable orthopedic implants. Further investigations are needed to examine the degradation behavior.

## Background

The first published application of a plate for fracture fixation was 1886 by Carl Hansmann [1]. It was not until the beginning of the 20^th ^century when plates as osteosynthesis systems was spread due to the works of William Arbuthnot Lane and Albin Lambotte. In 1907 Lane, a British surgeon, introduced perforated steel plates for use in internal fixation [2]. Lambotte was one of the first to apply pure magnesium plates in a clinical case to stabilize a fracture in a young man. After implantation he observed extensive subcutaneous gas cavities and local swelling caused by rapid degradation [3].

In recent years, new innovative magnesium alloys are being intensively investigated as potential resorbable materials with appropriate mechanical and corrosion properties. Particularly in orthopedic research magnesium is interesting because of its mechanical properties close to those of natural bone, the prevention of both stress shielding and removal of the implant after surgery [4]. Orthopedic implants need to provide an initial fixation and a predictable, gradually degradation adapted to the bone healing process.

ZEK100 is a magnesium alloy which contains 1 wt% zinc, 0.1 wt% zirconium and 0.1 wt% rare earth metals [5]. Magnesium and its degradation products are non-toxic and it is an essential co-factor for many enzymes; especially for DNA replication and repair processes [6,7]. Furthermore, magnesium acts as a stabilizer of DNA and chromatin structure. The recommended daily dietary is approximately 300 mg for adults [6]. Zinc as an alloying ingredient contributes to strength due to solid solution strengthening [7]. In addition, zinc is one of the essential micro-nutrients in the human body with an estimated daily requirement of 15 mg. It is essential to structure and function of over 300 enzymatic reactions [8]. The zinc finger motif, which is determined by a single zinc ion in the base, is most frequently occurring in transcription factors [8]. Zirconium is usually used as a grain refinement agent in magnesium alloys without aluminum and thereby contributes to strengthening [7]. In an in vitro study with binary magnesium alloys an addition of Zn or Zr showed good cytocompatibility, improved strength and a reduced corrosion rate [9]. The rare earth metals comprise seventeen elements which can be classified into two groups: elements with high and limited solubility in magnesium [10]. Rare earth (RE) metals improve strength by solid solution strengthening and precipitation strengthening [7]. The in vitro test with Mg-Y indicate no significant cytotoxicity to osteoblasts; furthermore good cytocompatibility was observed with Mg-Zn-Y alloys [9,11]. Several RE containing alloys such as WE43 or LAE443 have been investigated in former studies and reported good biocompatibility in vivo [12-16].

The disadvantage of magnesium alloys is the production of hydrogen gas which accumulates in tissue cavities. The amount of gas production is dependent on the corrosion rate. The accumulation of gas of ZEK100 implants was found in the medullary cavity via μ-computed tomography (μCT) examinations, though it did not became clinically visible [17].

In the present study, ZEK100 plates were examined in an in vitro model with Hank's Balanced Salt Solution under physiological conditions. A constant laminar flow of the corrosion medium was adjusted. The mechanical properties of the plates after corrosion were determined via four-point bending test. The surfaces of the immersed specimens were characterized by SEM, EDX and XRD.

## Methods

### Material

**Figure 1:**
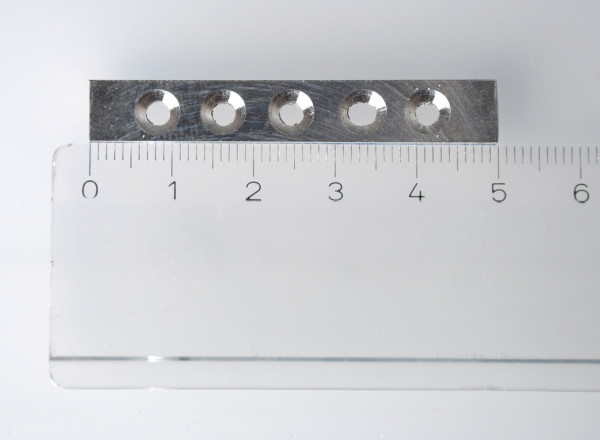
**Picture of a non-corroded plate**. The plate consists of ZEK100 and has a dimension of 50 mm × 8 mm × 1 mm. In every plate five screw holes were drilled and counterbored with an inner diameter of 3 mm on the bone-side and a counterbore diameter of 5 mm.

In this study plates for bone fixation of a biodegradable magnesium alloy were examined. The magnesium alloy ZEK100 contains 1 wt% zinc, 0.1 wt% zirconium and 0.1 wt% rare earth metals [5]. The analyzed chemical composition of the alloy by ICP-OES is given in Table 1. The ZEK100-specimens were cut into plates with dimensions of 50 mm × 8 mm × 1 mm by milling processes. In every plate five screw holes were drilled and counterbored. These screw holes have an inner diameter of 3 mm on the bone-side and a counterbore diameter of 5 mm (Figure 1).

**Table 1:** Nominal and analyzed composition of the ZEK100 alloy

Element	Mg	Zn	Zr	Rare Earth Elements			
				Y	Nd	Ce	La
Analyzed composition	98.53	0.961	0.211	0.146	0.02	0.092	0.042
Nominal composition	98.8	1	0.1		0.1		

### Microstructural characterization

The corroded plates were analyzed by a scanning electron microscope (SEM) EVO 60 VP by Zeiss. Theses analyses also included an energy dispersive X-ray analysis (EDX) to detect the distribution of elements on the surface of the corroded specimens and on the cross-section area of the plates. Therefore the plates were cut in the section of the middle bore. To achieve an electro conductive surface for the SEM analysis the specimens were also sputtered with gold.

X-ray diffraction (XRD) investigations were performed with a STOE Theta-Theta diffractometer in reflection geometry (wavelength *λ *= 1.54059 Å). The 2*θ *range from 2 to 65° was scanned with a step size of 0.01° (3.0 s per step) using monochromatized Cu Kα1 radiation.

### Immersion test in a system with a constant flow rate

**Figure 2:**
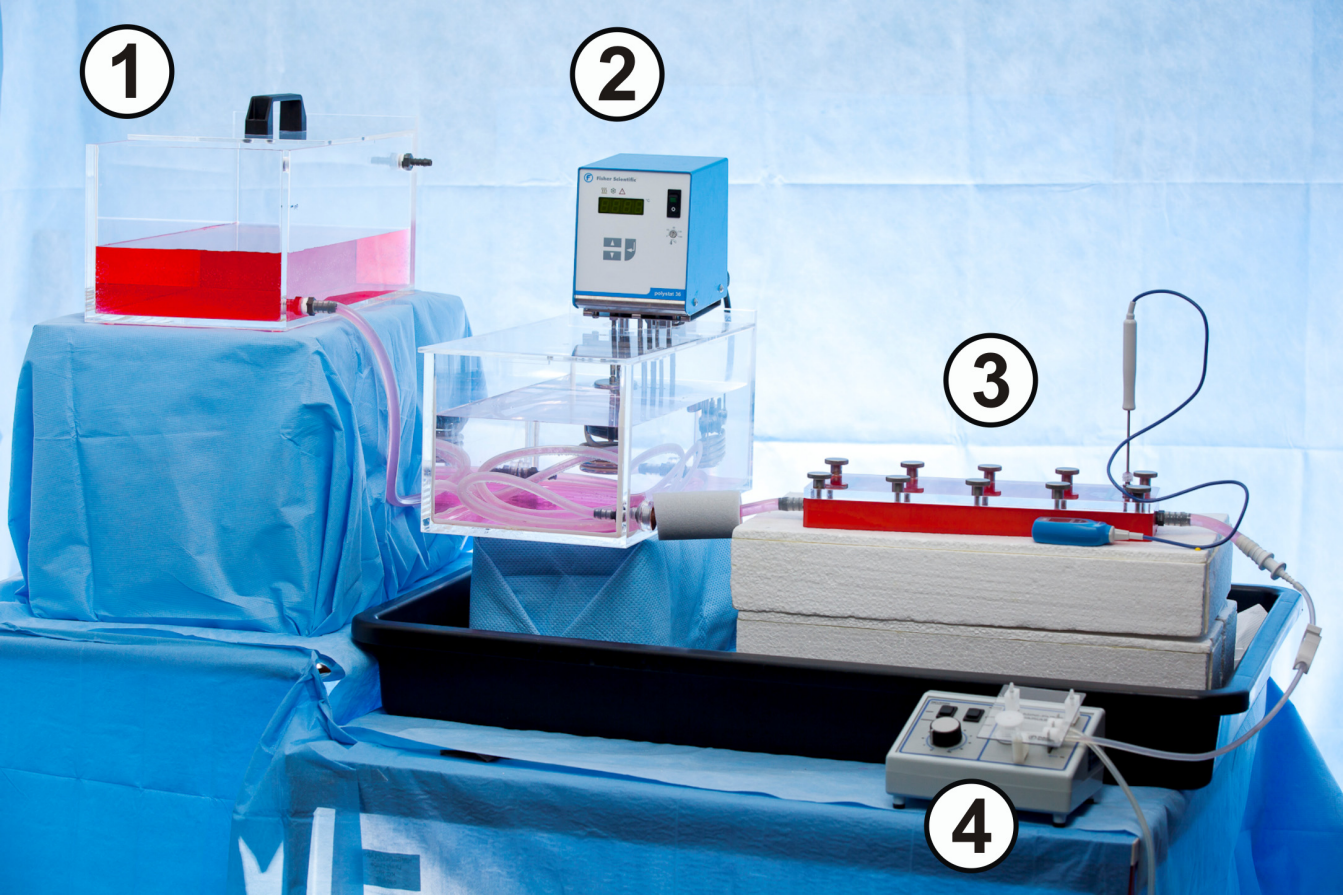
**Picture of the in vitro corrosion testing setup**. **1**: Glass basin with Hank's Balanced Salt Solution. **2**: The corrosion medium was warmed in a water bath to 37.4 +/- 0.5°C. **3**: HBSS passes the test system with the specimens with a laminar flow rate of 2.5 to 3.5 ml/min Temperature is checked with a thermometer. The system is protected against heat loss with styrofoam. **4**: The flow rate was maintained with a pump. The solution was then discarded in a container.

The immersion test was carried out in Hanks' Balanced Salt Solution (HBSS) to simulate normal ion concentration under physiological tissue conditions. The chemical composition of HBSS is given in Table 2. The HBSS were produced as dry substance with phenol red by Biochrom AG, Berlin. The temperature in the test system was measured by a thermometer (Fisher Scientific, Schwerte, Germany) during corrosion every day and regulated to 37.4°C +/- 0.5°C. Also the pH was controlled every day during corrosion and remained at 7.4. Flow rate was regulated to 2.5 to 3.5 ml/min. After passing the ZEK100 alloys the solution was discarded (Figure 2).

**Table 2:** Chemical composition of Hanks' Balanced Salt Solution

Reagents	Concentration (mg/l)
NaCl	8000
KCl	400
Na_2_HPO_4_	48
KH_2_PO_4_	60
MgSO_4_·7H_2_0	200
CaCl_2_	140
Glucose	1000
Phenol red	10
NaHCO_3_	350

In this study twelve ZEK100 plate demonstrators, divided into 3 groups with 4 demonstrators, were corroded and tested. The demonstrators were fixed into a test frame which allows a laminar flow of the HBSS. The first group was corroded over 14 days, the second group over 28 d and the third group over 42 days.

### Mechanical testing

**Figure 3:**
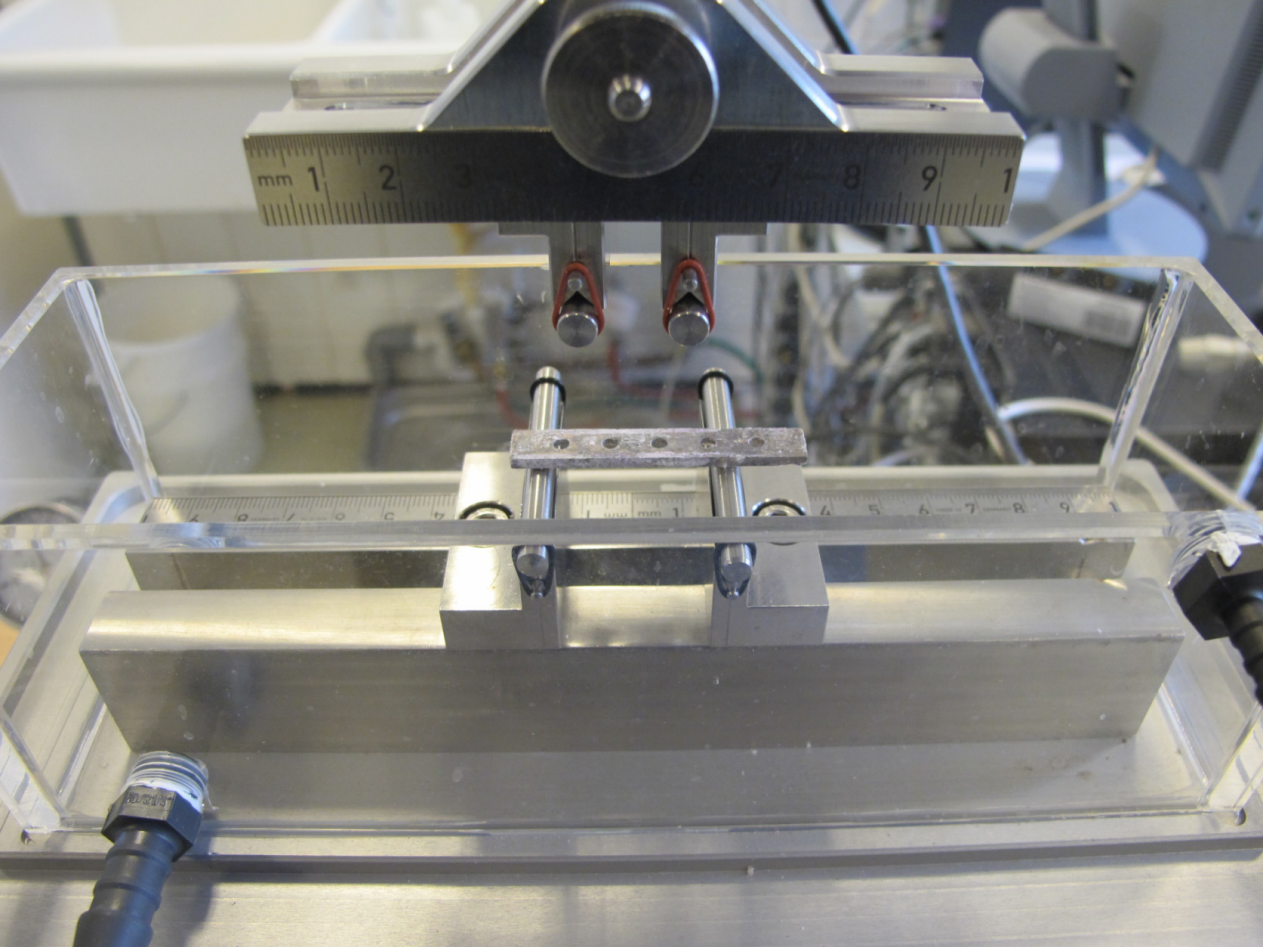
**Picture of the mechanical four-point bending test setup**. The test system consisted of four rollers with a diameter of 8 mm. The distance between the lower and outer rollers was 32 mm. 10 mm distance was between the upper and inner rollers. The plates were positioned on the lower rollers according to the protocol of ISO 9585:1990.

In order to specify the mechanical properties of the specimens, they were tested according to ISO 9585:1990. The four point bending test setup (Figure 3) was designed for tests with an uniaxial material-testing machine (Mini Bionix 858, MTS Systems in Mineapolis, USA). The test system consisted of four rollers with a diameter of 8 mm. The distance between the lower and outer rollers was 32 mm. 10 mm distance was between the upper and inner rollers. The plates were positioned on the lower rollers according to the protocol of ISO 9585:1990. The distance between the outer and inner rollers was 11 mm and contains a screw hole of the demonstrators. The test rig was produced by the Research Work Shop of the Medical School, Hannover, Germany.

## Results

### Microstructure and surface composition after immersion

**Figure 4:**
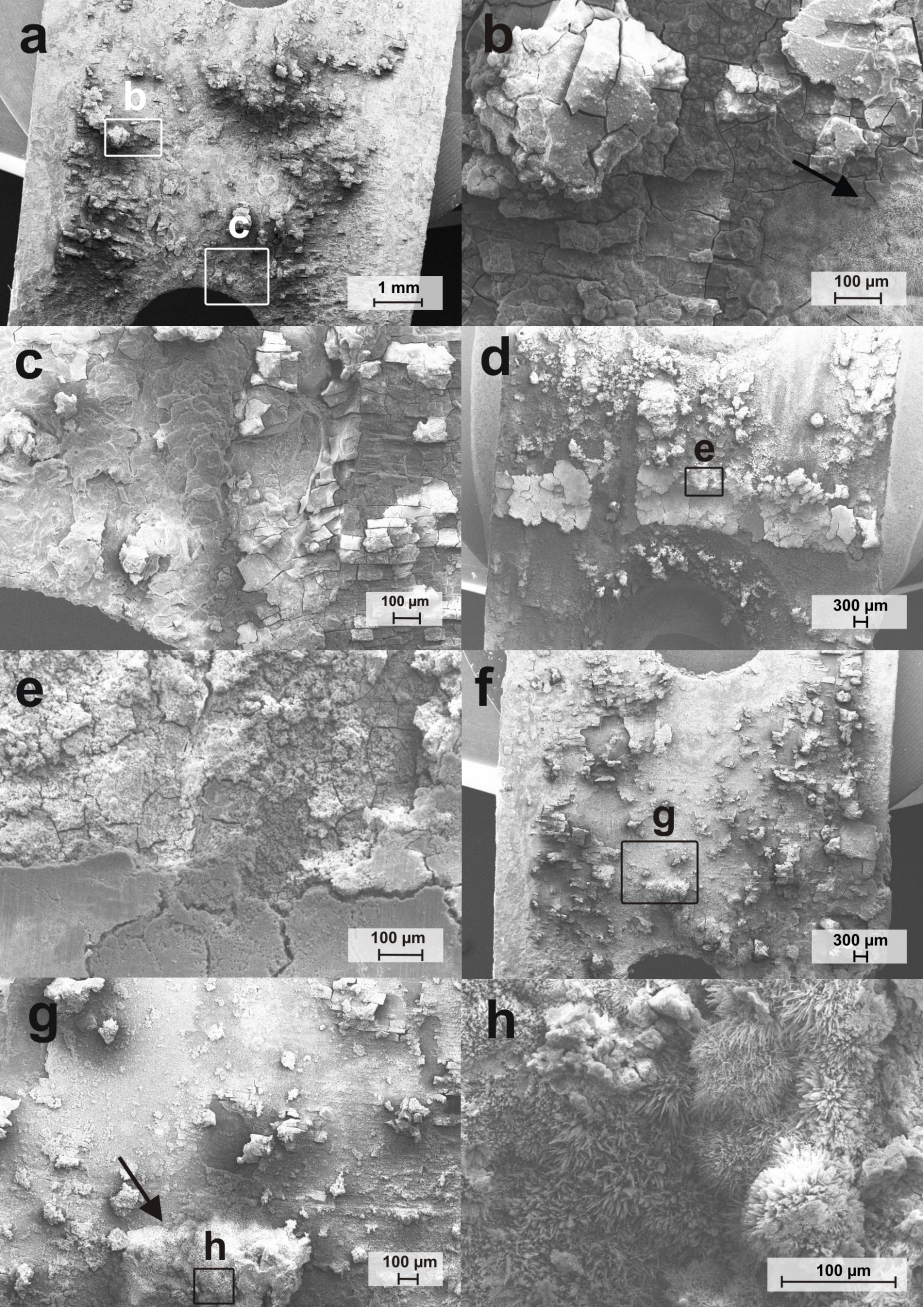
**SEM pictures of the surface morphology of ZEK100 plates immersed in Hank's balanced salt solution**. a)-c) after 2 weeks, d)-e) after 4 weeks, f)-h) after 6 weeks. The black arrow marks needle-shaped clusters.

**Figure 5:**
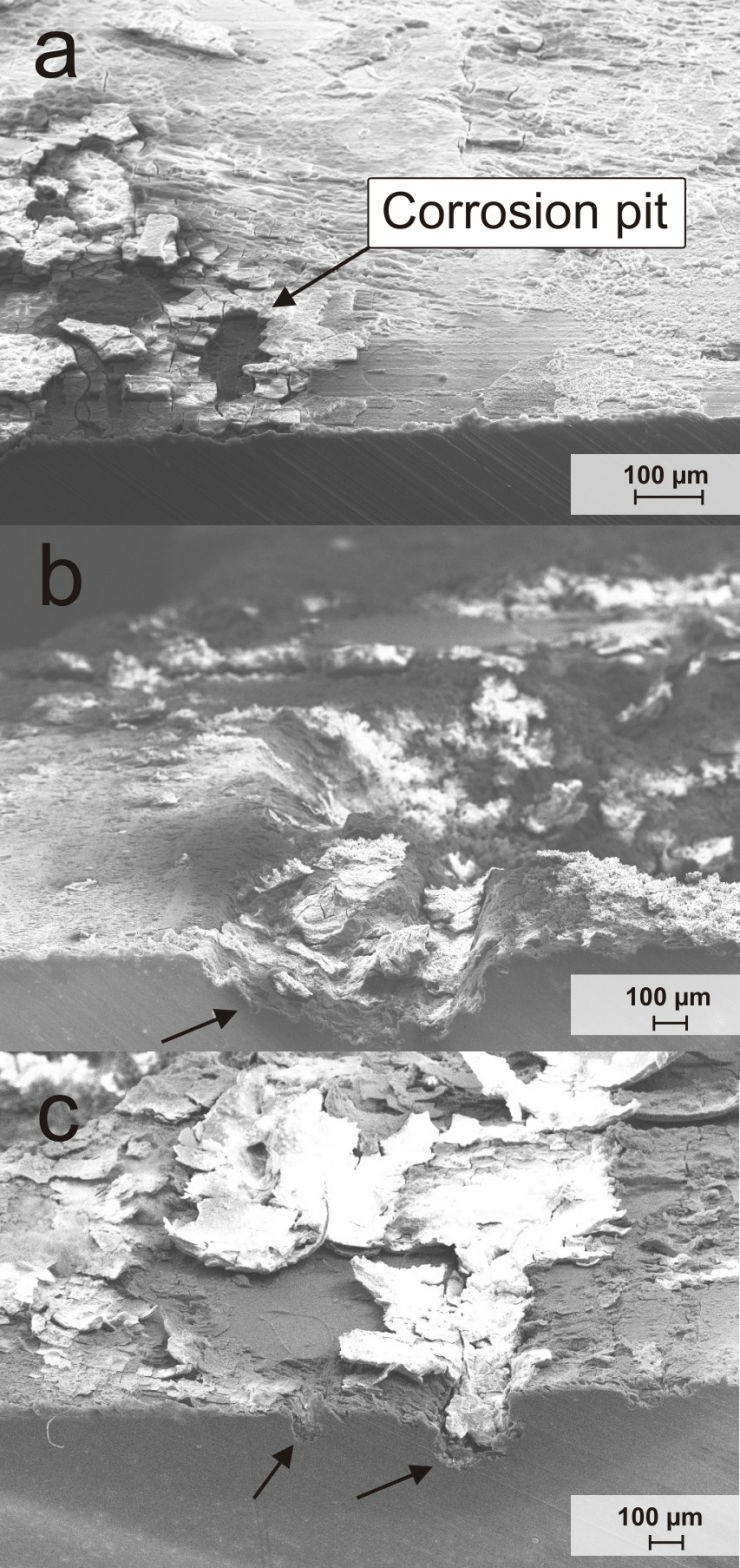
**SEM pictures of the cross-section area**. a) 2 weeks, b) 4 weeks c) 6 weeks. The corrosion pits are marked by black arrows.

The surface morphology of ZEK100 plates after exposure in Hank's Balanced Salt Solution are shown in the SEM images of Figures 4 and 5. On the corrosion surface cracks were found after all immersion intervals which might be caused by drying. At higher magnifications, some needle-shaped clusters appeared on the surface of the 2 weeks group and the 6 weeks group (Figure 4b, g). A more detailed picture of that structure is given in Figure 4h. In the SEM picture after 4 weeks immersion a peeling of corrosion surface can be observed in high magnification view. Small, agglomerated particles seemed to be formed on the surface (Figure 4e). The pictures of the cross-sections reveal local pits on the surface (Figure 5). More distinct and deep pits were found after 4 and 6 weeks immersion (Figure 5b, c).

**Figure 6:**
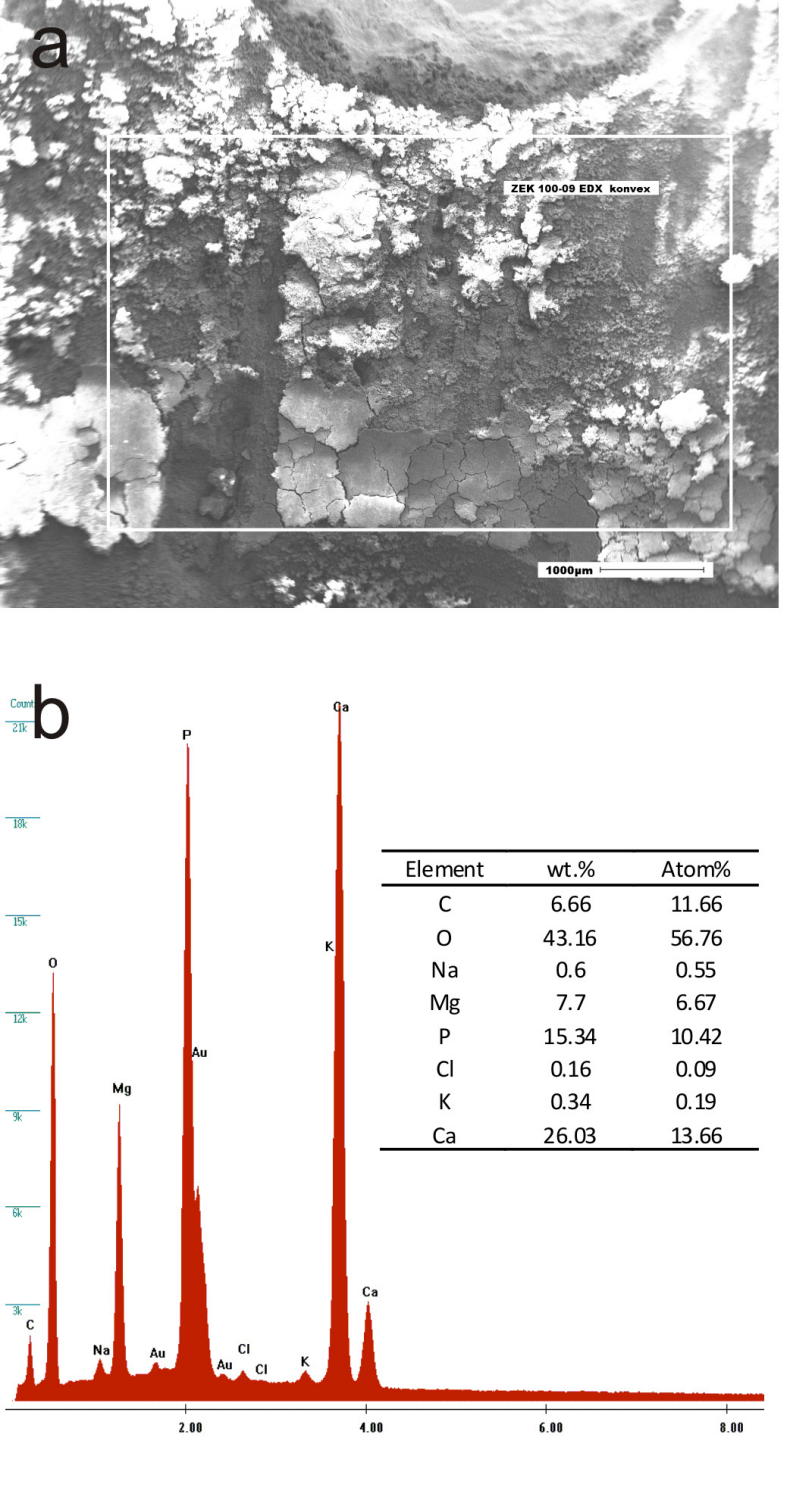
**Example of the EDX analysis**. The energy dispersive X-ray analysis (EDX) was applied in order to detect the distribution of elements on the surface of the corroded specimens.

EDX analyses were conducted on a small surface area after every corrosion interval to determine the elemental composition (Figure 6). The results revealed the presence of high amounts of the elements O, P and Ca and small amounts of C, Na, Mg, Cl and K (Table 3).

**Table 3:** Results of the EDX analysis of the ZEK100 alloy

Element	2 weeks		4 weeks		6 weeks	
	**wt%**	**at%**	**wt%**	**at%**	**wt%**	**at%**
C	6.21	10.8	6.66	11.66	6.3	11.01
O	43.07	56.24	43.16	56.76	42.3	55.48
Na	0.63	0.57	0.6	0.55	1.94	1.77
Mg	10.88	9.35	7.7	6.67	10.36	8.94
P	16.94	11.43	15.34	10.42	14.99	10.16
Cl	0.08	0.05	0.16	0.09	0.06	0.04
K	0.28	0.15	0.34	0.19	0.6	0.32
Ca	21.92	11.42	26.03	13.66	23.45	12.28

**Figure 7:**
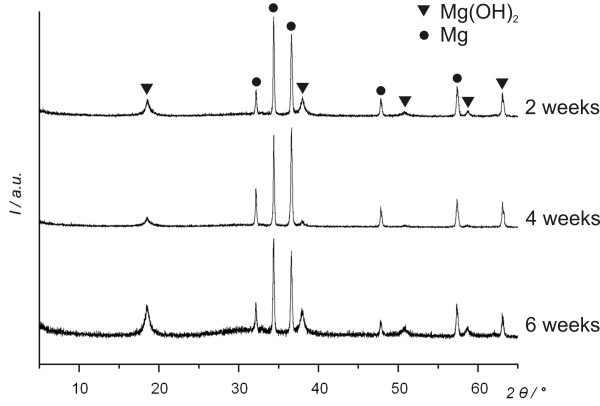
**X-ray diffraction (XRD) patterns of ZEK100 plates**. The XRD analysis of the plates after different immersion times in Hank's Balanced Salt Solution.

X-ray diffraction (XRD) was applied to identify some of the corrosion products on the surface. Figure 7 presents the XRD patterns of the ZEK100 plates after all three different immersion intervals. In all spectra large magnesium and Mg(OH)_2 _peaks are clearly visible.

### Mechanical characterization of the specimens after immersion

**Figure 8:**
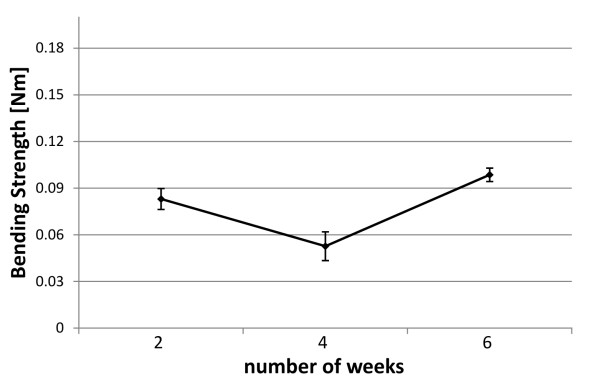
**Bending strength of ZEK100 plates**. The bending strength of the plates after different time intervals of exposure to Hank's Balanced Salt Solution.

The bending strength was calculated from obtained load-deflection curves. The results are presented in Figure 8. The results of the bending test displayed a decrease of bending strength from immersion week 2 to immersion week 4. The tested plates after 6 weeks corrosion showed an increased bending strength.

## Discussion

Fracture healing is a complex pathway with determine steps and the healing quality and speed depends on the size of the fracture gap and the achieved stability. Therefore an adjusted stability is essential for fracture healing. The discrepancy of the Young's modulus of the conventional implants (stainless steel or titan) in regard to bone promotes the occurrence of stress shielding with negative effects to the bone healing. Degradable implants from magnesium alloys are desirable because of the mentioned promising characteristics of magnesium. Standard tests with those implants according to ASTM protocols are rare.

In the present study, ZEK100 plates were examined in an in vitro model with Hank's Balanced Salt Solution under physiological conditions. The corrosion medium used in this study is in its ionic content similar to blood plasma with a high chloride concentration. The degradation of magnesium and its alloys are described according to the following reactions [18]:

Anodic reaction: Mg → Mg^2+ ^+ 2e^-^

Cathodic reaction: 2 H_2_O + 2e^- ^→ H_2_↑ + 2 OH^-^

$$\text{M}{\text{g}}^{2+}+\text{ 2 O}{\text{H}}^{\text{ - }}\to \text{Mg}{\left(\text{OH}\right)}_{2}\left(\text{s}\right)$$

It is reported that the protective Mg(OH)_2_-film is formed rapidly after 2 h immersion [19]. The Mg(OH)_2_-layer on the surface is dissolved by Cl^- ^into soluble MgCl_2_:

$$\text{Mg}{\left(\text{OH}\right)}_{2}\to \text{MgC}{\text{l}}_{2}+\text{ 2 O}{\text{H}}^{-}$$

**Figure 9:**
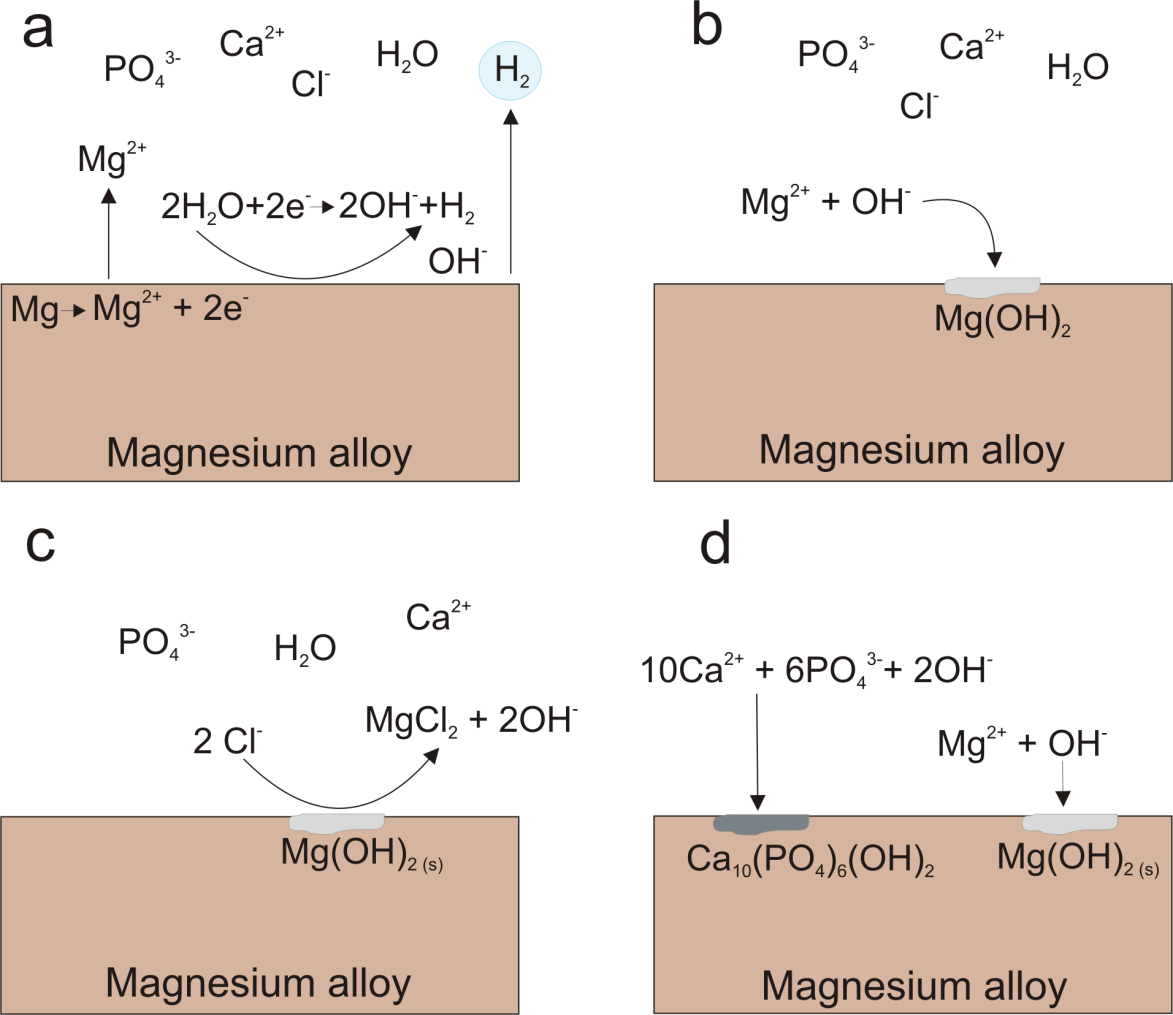
**Schematic diagram of the reactions on the surface of the magnesium alloy**. a) The galvanic reaction between magnesium and H_2_O, b) the formation of the magnesium hydroxide film on the surface, c) the transformation of Mg(OH)_2 _into soluble MgCl_2_, d) the formation of hydroxyapatit on the surface due to consumption of Ca^2+ ^and PO_4_^3-^.

An illustration of the reactions on the surface of the magnesium alloy is given in Figure 9. This diagram is in accordance with previous illustrations in Ref. [20,21] and is intended to demonstrate the corrosion processes on the surface. This dissolution reaction elevates the local hydroxide concentration near the surface. It is reported that in stagnant corrosion systems the degradation of magnesium and its alloys lead to an alkalization of the corrosive medium [22]. As a result of alkalization, a greater tendency for film formation is reported when the local pH value rise above 10.5 [23]. However, this effect might be minor in this study due to the NaHCO_3 _buffer in the corrosion medium and the flow rate applied in this system. Nevertheless, it is reported that this buffering agent accelerates the dissolution of magnesium because of the consumption of the generated OH^- ^[24].

The corrosion in our model is most likely more influenced by the velocity. The accelerating corrosive effect of the flow rate obtains to (i) shear forces, (ii) the destruction or prevention of a protective surface layer and (iii) the prevention of micro-alkalization on the surface [7,23]. It is noticeable that a corrosion layer could be identified considering the flow rate adjusted in this immersion model. Wang et al. compared the degradation rate of a dynamic system with a flow rate to static conditions with Hank's Balanced Salt Solution by weight loss method [25]. An accelerated degradation rate was reported in this study under dynamic conditions; though the applied flow rate remains unclear [25].

The EDX analysis of our plates revealed a high amount of the elements Ca, P and O which indicates the precipitation of calcium phosphates with low solubility which was reported in previous studies [15,18,26]. The corrosion medium Hank's Balanced Salt Solution contains a certain amount of Ca^2+ ^and PO_4_^3- ^(Table 1). The most stable calcium phosphate is hydroxyapatite which forms according to the following reaction:

$$\text{1}0\text{ C}{\text{a}}^{2+}+{\text{ 6 PO}}_{4}^{3-}+\text{ 2 O}{\text{H}}^{-}\to \text{C}{\text{a}}_{10}{\left(\text{P}{\text{O}}_{4}\right)}_{6}{\left(\text{OH}\right)}_{2}\left[\text{27}\right].$$

Additional types of calcium phosphates are in order of increasing solubility: Ca_2_(PO_4_)_3_·nH2O (TCP), Ca_8_H_2_(PO_4_)_6_·5H2O (OCP) and Ca(HPO_4_)·5H_2_O (DCPD) [27]. Hydroxyapatite is an essential component of the human bone and therefore the biocompatibility of this precipitation layer can be assumed. In addition, it has been shown that hydroxyapatite coated carboxymethyl chitosan (CMCS) enhances the proliferation and differentiation of osteoblasts and stem cells [28]. In a different study Ca_3_Mg_3_(PO_4_)_4 _was detected on the surface via XRD analysis of a Mg-Mn-Zn alloy immersed in Hank's solution [18]. The occurrence of this specific precipitation layer in our study is not supported by the low Mg content in our EDX analysis. The precipitation layers protect the magnesium alloy against aggressive ions in the corrosive medium and therefore reduce the corrosion rate [18]. In corrosive media containing HCO_3_^-^, which is the case in HBSS and blood plasma, the surface layer might consist of amorphous carbonated calcium phosphate with a schematic formula as follows: (Mg, Ca)_x_(PO_4_)_y_(CO_3_)_z_(OH)_i _[29].

An advanced deposition of calcium and phosphate on the surface was determined by EDX analysis in all specimens. These results are comparable with Huan et al., who investigated an Mg-Zn-Zr alloy which is comparable to ZEK100 [30]. In this mentioned study the atomic content of Mg decreased slightly from 7 days immersion to 24 days while the atomic content of P and Ca increased [30]. The growth of the corrosion precipitation layer could not be detected in this study by EDX analysis after the different immersion time intervals. This let us assume that after 2 weeks the composition on the surface is stable which might be explained by an equilibrium between precipitation and erosion.

The effect of corrosion on the bending strength of plates with medical geometry has not been studied previously. In the present work a decreased bending strength of the plates was determined after 4 weeks in Hank's Balanced Salt Solution in comparison to the 2 week and 6 week group. It can be concluded that the change of the bending strength after 4 week and 6 week immersion might be caused by the embrittlement of the magnesium alloy. As described hydrogen gas evolve as bubbles from the magnesium surface in consequence of dissolution. It is also reported that some hydrogen atoms enter the metal lattice [31,32]. This storage of hydrogen atoms results in hydrogen embrittlement of the magnesium alloy which degree is dependent on the immersion time [31]. It is reported that the cohesive strength of a magnesium alloy (AZ31) is reduced by hydrogen embrittlement [31].

In a previous in vivo study the bending stiffness of ZEK100-implants was determined via 3 point bending test. The maximum force (F_max_) at the point of breakage of the explanted implants was decreased by 35% after 3 months and 60% after 6 months. These in vivo results differ from the results determined in this study. The geometry of the implant affects the determination of the mechanical properties of the specimen. In the in vivo study by Hühnerschulte et al. pins were used as implant geometry [17]. Further in vivo evaluations with the dimensions examined in this study would be helpful to improve the comparability between the in vivo and in vitro corrosion behavior. Different biomechanical results may be caused by different corrosion rates in vitro and in vivo [33,34]. It is reported that the in vitro corrosion rate is faster than in comparable in vivo studies [33]. Müller et al. reported that the corrosion rate was in vitro 1.7 times greater than in vivo for LAE442 [33]. Faster corrosion rates should be accompanied with an earlier loss of mechanical strength. A rapid decrease of the bending strength was determined from week 2 to week 4 in this in vitro study.

In contrast to this, in vivo μCT scans first demonstrate a decrease of the implant volume until week 4, thereupon an increased volume was determined up to week 8. After week 8 a continuously reduction of the volume was observed [17]. This increase of implant volume could be explained by the corrosion products on the surface which is not differentiated by the contour [17]. We assume that this increase of the volume caused by precipitation in the first weeks of corrosion affects the bending strength of the implants at that time. In our study the precipitation of calcium phosphates on the surface was observed via EDX analysis. This might explain the higher bending strength in week 6.

Primary bone healing is induced by a stiff construct and secondary bone healing by limited passive or active dynamization. However the role of mechanical stability in the fracture healing process is not totally cleared yet. Therefore it is not desirable to produce a total rigid complex. A mechanical stimulus is essential for bone growth and consolidation. It is still controversial which tactic would best improve the duration and strength of the healing fracture. But it is clear that stress shielding occurs as a result of the different Youngs moduls. The mention benefit of magnesium based implants is the low Youngs modul, this could avoid stress shielding.

There is a discrepancy between the maximum strength and the optimum strength. Magnesium plates are able to offer a time depending stability. However, it is important to define the demanded stability of a specific fracture. The needed stability of fracture depends on the bone quality, fracture geometry, localization of the fractured bone and the demand in rehabilitation. Therefore, the first demand should be define. According to the required implant characteristics the test standards should also be adjusted.

## Conclusion

In the present work the degradation of ZEK100-plates was investigated. The bending strength increased after 6 weeks immersion compared to the 2 week group and 4 week group. The characterization of the surface revealed the presence of high amounts of O, P and Ca on the surface and small Mg content. This indicates the precipitation of calcium phosphates with low solubility on the surface of the ZEK100 plates. The present in vitro study indicates that ZEK100 is a potential candidate for degradable orthopedic implants. Further investigations are needed to examine the degradation behavior.

## Abbreviations

EDX: Energy dispersive X-ray analysis; HBSS: Hank's Balanced Salt Solution; ICP-OES: Inductively coupled plasma optical emission spectrometry; μCT: μ-computed tomography; RE: Rare earth; SEM: Scanning electron microscope; wt%: Weight percent; XRD: X-ray diffraction; ZEK100: Magnesium alloy with 98.8 wt% magnesium 1 wt% zinc, 0.1 wt% zirconium and 0.1 wt% rare earth metals

## Competing interests

The authors declare that they have no competing interests.

## Authors' contributions

HW supervised the in vitro corrosion examinations, analyzed the data and prepared the manuscript. AW analyzed the data and wrote the manuscript. CM performed the in vitro corrosion examinations and wrote the manuscript. FW and HW participated in the coordination of the corrosion experiments and helped to draft the manuscript. AL manufactured the plates and analyzed them via SEM and EDX. MK did the XRD analysis of the ZEK100 plates. BD, PB, AML and FWB revised the manuscript primarily although all authors made contributions. FT initiated the study, participated in its design and coordination. All authors read and approved the final manuscript.
